# Closure of subarterial ventricular septal defect with minimally invasive surgical technique: A case report

**DOI:** 10.1016/j.ijscr.2019.04.025

**Published:** 2019-04-16

**Authors:** Duc Hung Duong, Quoc Dat Pham

**Affiliations:** Vietnam National Heart Institute, Bach Mai Hospital, 78 Giai Phong, Dong Da, Hanoi, Viet Nam

**Keywords:** Subarterial ventricular septal defect, Minimally invasive cardiac surgery, Left parasternal thoracotomy, Case report

## Abstract

**Introduction:**

Minimally invasive cardiac surgery has been applied for the treatment of ventricular septal defect (VSD) with various approaches. However, closure of subarterial VSD with minimally invasive technique via left parasternal thoracotomy is rarely reported.

**Case presentation:**

A 22-year-old man, weighing 65 kg, with a diagnosis of subarterial VSD underwent successful repair with minimally invasive technique via left parasternal thoracotomy through third intercostal space. The peripheral perfusion was performed with femoral arterial and venous cannulation. Myocardium was protected by warm blood cardioplegia injected directly into aortic root by a long needle and aortic clamp introduced through the thoracotomy incision.

**Discussion:**

The left parasternal thoracotomy through third intercostal space (ICS) allows to expose both the subarterial VSD and ascending aorta. Myocardial protection and repair of this defect can be performed merely without requirements of video assistance or unique instruments. The patient recovered rapidly and was satisfied with the cosmetic result. The primary concern of this technique is mammary tissue which can be injured by a transverse incision in female patients. In this case, we can transform into the longitudinal incision.

**Conclusion:**

This minimally invasive technique is feasible for the surgical treatment of subarterial VSD. Long-term follow-up and additional cases will be needed for validation of the safety and efficacy of this approach.

## Highlights

• Minimally invasive cardiac surgery for closure of subarterial ventricular septal defect.
• The approach using left parasternal thoracotomy via third intercostal space.
• Excellent exposure of subarterial ventricular septal defect without special instruments.

## 1 Introduction

Subarterial type VSD has a high prevalence in the Asian population, accounting for about 30% of all VSDs [1]. Early intervention is recommended due to this defect is closely related to progressive aortic regurgitation, and spontaneous closure is uncommon. Because of its proximity to the aortic valve, device closure of subarterial VSD is challenging to perform. Therefore, surgical closure is indicated in most cases [2]. The standard approach for repair of VSDs is conventional median sternotomy which allows for good exposure of the surgical field and safe closure of this defect. However, limitations of this incision are undesirable cosmetic results and sternotomy-related complications [3]. A variety of alternative approaches described, such as a partial sternotomy, thoracotomy or totally thoracoscopy have been applied to reduce invasiveness and improve cosmetic results.

In this paper, we described the minimally invasive technique for closure of subarterial VSD via left parasternal thoracotomy through third intercostal space. The work has been reported in accordance with the SCARE criteria [4].

## 2 Case report

A 22-year-old man, weighing 65 kg, was admitted to our hospital to evaluate a murmur in the routine examination. He had complained of mild shortness of breath on physical exertion for one month. Physical examination on admission revealed a systolic murmur in the left para-sternum, trans-thoracic echocardiography showed a subarterial VSD with a diameter of 8 mm, left to right shunting, pressure gradient via the defect was 70 mmHg. The left ventricle dilated mildly with left ventricle end-diastolic diameter was 57 mm and left ventricular ejection fraction was normal range. Pulmonary artery systolic pressure was 33 mmHg at rest. Mild aortic regurgitation was present.

**Fig. 1:**
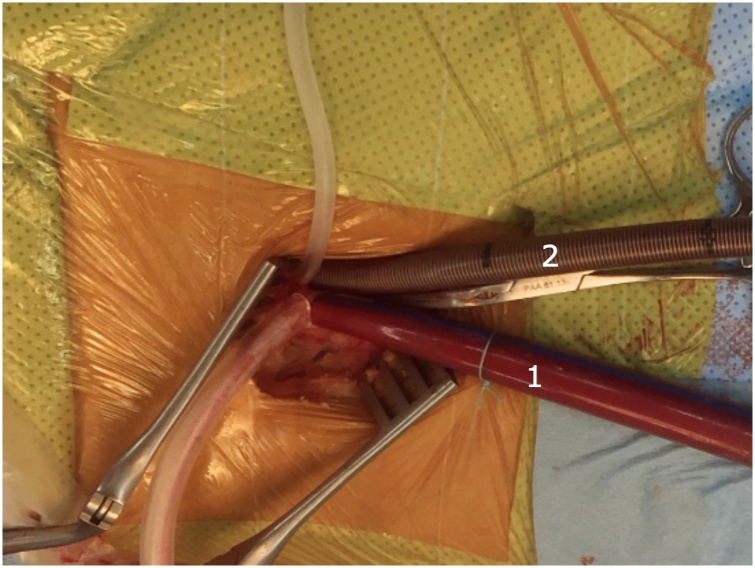
Peripheral cannulation. The femoral artery cannula was inserted directly into the common femoral artery. A multi-stage venous cannula was inserted using the Seldinger technique with the tip of cannula advanced to the superior vein cava under transesophageal echocardiography guidance. (1): arterial cannulation, (2): venous cannulation.

**Fig. 2:**
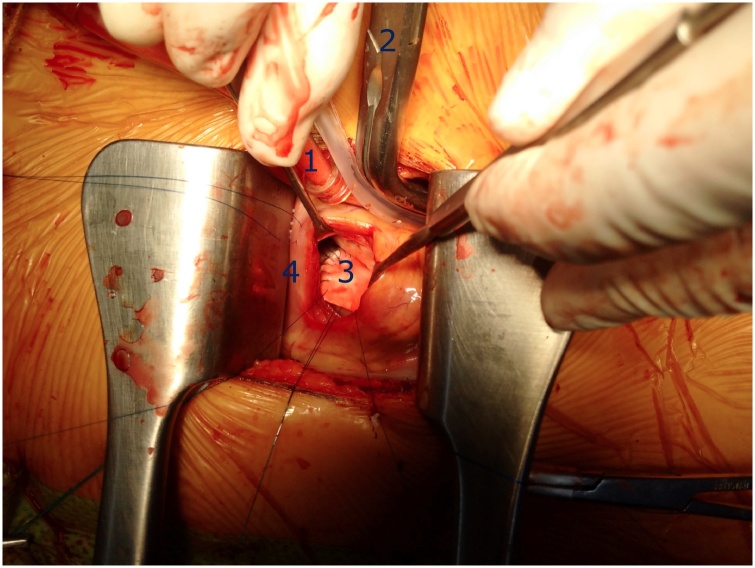
Left parasternal thoracotomy via 3rd intercostal space. Myocardium was protected by warm blood cardioplegia injected directly into aortic root by a long needle and aortic clamp introduced through the thoracotomy incision. Subarterial VSD was exposed through a transverse right ventriculotomy and closed with a patch by continuous suture. (1): long cardioplegia needle, (2): aortic clamp, (3): patch for closure of VSD, (4): transverse right ventriculotomy.

**Fig. 3:**
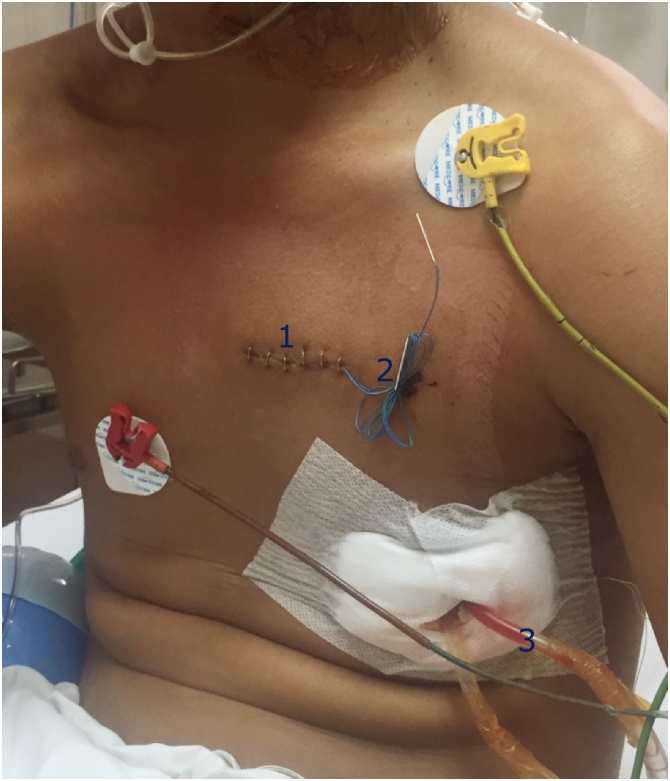
Postoperative images. (1): surgical scars at 3rd intercostal space, (2): temporary epicardial pacing electrodes, (3): pericardial and left pleural drains.

The patient was conducted under general anesthesia with a single-lumen endotracheal tube and placed in supine position as for standard median sternotomy with two arms along the body. Defibrillation pads were placed on the right and left chest before sterile draping. The femoral artery and vein were dissected in preparation for cannulation with a 2–3 cm oblique right groin incision. A 4 cm left parasternal thoracotomy was used to enter the thorax via the third intercostal space (ICS). The left internal thoracic artery was preserved carefully. The third costal cartilage was divided close to the sternum, without resection, to increase exposure. The ribs were slowly spread with a mini-thoracic retractor. The pericardium was opened longitudinally and suspended with stay sutures. The femoral artery cannula was inserted directly into the common femoral artery. A multi-stage venous cannula was inserted using the Seldinger technique with the tip of cannula advanced to the superior vein cava under transesophageal echocardiography guidance. After femoral arterial and venous cannulation, cannulas were secured, and cardiopulmonary bypass (CPB) initiated. CPB was initiated with vacuum-assisted venous drainage and body temperature maintained at approximately 34 °C A long cardioplegia needle (Livanova, London United Kingdom) was utilized to deliver warm blood cardioplegia directly into the aortic root and repeated every 15–20 minutes. An aortic clamp was introduced through the thoracotomy incision as a standard median sternotomy. The aorta was cross-clamped after dissecting the main pulmonary artery from ascending aorta. The VSD was exposed through a transverse right ventriculotomy. A left vent was inserted via the VSD to evaluated the edge of the defect. The subarterial VSD was closed with a patch (Bovine pericardial patch, Edwards Lifesciences) by a continuous suture. The left heart was filled with saline to exclude air before tying the suture. Any remaining air was then vented through the original cardioplegia site. Ventriculotomy was closed, and two ventricular temporary epicardial pacing electrodes were placed before releasing the aortic clamp. The cardioplegia needle was removed after the de-airing maneuver was completed, and the patient might be weaned from CPB and decannulated. CPB and cross-clamp times were 58 and 42 min, respectively. The patient was ventilated postoperatively in the intensive care unit and extubated within 4 h without any complications. Echocardiography prior to discharge showed completely closed VSD, mild aortic regurgitation. The patient was discharged from the hospital on the fifth postoperative day. There were no complications after 3 months and 6 months of follow-up (Fig. 1, Fig. 2, Fig. 3).

## 3 Discussion

Median sternotomy is considered the standard surgical approach for the repair of VSDs. With surgical advancements, the incidence rate of mortality from simple congenital heart diseases such as atrial septal defect or ventricular septal defect drops to near zero. In an attempt to improve cosmetic results and avoid the potential drawbacks associated with conventional sternotomy, various approaches have been applied such as a partial sternotomy, subaxillary thoracotomy, anterior thoracotomy or totally thoracoscopy [[5], [6], [7], [8], [9], [10], [11], [12]].

Using mini-sternotomy for surgical treatment of VSDs was reported by some authors [11], [12]. Firstly, this technique was applied for correction of atrial septal defect and then other congenital heart diseases, especially ventricular septal defect [10]. This approach is always feasible to convert to a full sternotomy. Central cardiopulmonary bypass and myocardial protection are performed efficiently, and surgical field is exposed adequately for repair of VSDs as well as other concomitant defects. Otherwise, the sternotomy-related complications remained drawbacks of this procedure.

An et al. reported 78 patients with doubly committed subarterial ventricular septal defects treated with minimally invasive surgical closure through a right subaxillary thoracotomy. The defect was closed through the main pulmonary artery or right ventricular outflow tract. The other concomitant defects, atrial septal defect, mitral valve or tricuspid valve also can be repaired with this approach. However, exposing the defect through the main pulmonary incision with right subaxillary thoracotomy is not always easy. Because patients are placed with the right side elevated 60°–75°, central cannulation is required in all patients. Thus, it is associated with a longer incision and larger spread of ribs [5].

Another minimally invasive technique for closure of VSD described by Lin et al. is the video-assisted left anterior thoracotomy via 4th ICS with peripheral cannulation. All patients underwent repair of VSDs through right ventriculotomy and recovered rapidly from the operation. The myocardium was protected by continuous coronary perfusion with hypothermic fibrillatory arrest in almost patients due to exposure of ascending aorta is difficult with this approach [6].

Some authors have described totally thoracoscopic techniques for closure of perimembranous VSD. The advantages of this method are cosmetic results and faster recovery. On the other hand, the drawbacks are the technical complexity and long-term learning curve. Moreover, performing myocardial protection and de-airing maneuvers are more difficult. Therefore, this approach often requires longer cross-clamp and CPB times [[7], [8], [9]].

Our left parasternal thoracotomy via 3rd ICS is similar to right parasternal thoracotomy in minimally invasive aortic valve surgery [13]. This incision allows excellent exposure of both the defect and ascending aorta. Effective myocardial protection and de-airing procedures were performed through the aortic root. Furthermore, when necessary, aortic cannulation can be inserted directly. The subarterial VSD can be repaired simply without requirements of video assistance or any special surgical instruments. However, the left mammary tissues can be injured by transverse skin incision in female patients. In that case, we can transform into longitudinal incision to avoid this damage. The patient recovered rapidly and discharged the hospital 5 days after the operation. The patient was satisfied with the cosmetic incision.

## 4 Conclusion

This minimally invasive technique is feasible for the surgical treatment of subarterial VSD. The approach through left parasternal thoracotomy via 3rd ICS provides another option for correction of this defect. Long-term follow-up and additional cases will be needed for validation of the safety and efficacy of this approach.

## Conflicts of interest

No conflict of interest declared

## Sources of funding

No funding was received for the study

## Ethical approval

Ethical approval is not needed in Vietnam

## Consent

Written informed consent was obtained from the patient for publication of this case report and accompanying images. A copy of the written consent is available for review by the Editor-in-Chief of this journal on request.

## Author contribution

Both authors, Dr. Duong and Dr. Pham have taken part in conception of the study, drafting and revising the whole manuscript critically. All authors have given their final approval of the manuscript upon submission.

## Registration of research studies

None.

## Guarantor

Dat Q. Pham.
